# Cloth versus disposable diapers: an exploratory study on family habits

**DOI:** 10.1016/j.jped.2024.10.008

**Published:** 2024-12-09

**Authors:** Marjorie Uber, Renata R. Imoto, Vânia O. Carvalho

**Affiliations:** Universidade Federal do Paraná (UFPR), Departamento de Pediatria, Divisão de Dermatologia Pediátrica, Curitiba, Brazil

**Keywords:** Diapers, Diaper rash, Cloth diaper, Disposable diaper, Dermatitis, Contact dermatitis

## Abstract

**Objective:**

To describe features and habits of diaper area care and compare the frequency of diaper dermatitis in infants using cloth diapers with those using disposable diapers.

**Methods:**

Questionnaires were administered to families with infants who had not started potty training, to assess the frequency of diaper rash in two groups: babies who use exclusively cloth diapers (CD), and others with exclusively disposable diapers (DD). The hygiene methods of the perineal region and the skin lesions frequency were evaluated. The study was approved by the Ethics Committee.

**Results:**

1389 participants were included, 53 % male, with a median age of 16 (7–24) months, 1269 (91.4 %) in DD and 120 (8.6 %) in CD. Mild diaper rash occurred a few times a year in 47.0 % and 47.5 % in the DD and CD groups, respectively (*p* = 0.47). Severe diaper rash occurred a few times a year in 13 % and 10.7 % in the DD and CD groups, respectively (*p* = 0.66). In the DD, the most used hygiene method was wet wipes (61.5 %), whereas in the CD it was cotton/cloth with water (62.2 %; *p* < 0.001).

**Conclusion:**

Disposable diapers continue to be more used; hygiene habits differ between the groups and the use of cloth diapers did not increase the frequency of diaper dermatitis when compared to the use of disposable diapers.

## Introduction

One of the challenges experienced by families is the occurrence of dermatitis in the diaper region, which can be caused by primary irritant dermatitis, allergic contact dermatitis and fungal infections. The former is the most common and its main trigger is the contact of urine and feces with the skin,^1^ so adequate cleaning, frequent changes and the use of absorbent diapers that minimize skin contact with excreta are essential to avoid changes to the skin barrier in this region, which culminates in diaper dermatitis.^2^

Diapers made of different materials have been part of global culture for years.^3^ Disposable diapers are the most used in most countries, but they generate a large volume of non-compostable and non-biodegradable waste.

Cloth diapers have been adopted by many families around the world, whether for environmental or health reasons. In the past, reusable diapers caused more severe and frequent irritant dermatitis in the diaper area.^4^ However, with the technological evolution of fabrics and models, it is possible that this paradigm has changed.

There is a lack of comparative studies on different types of diapers and their impact on the frequency of diaper dermatitis. Therefore, doctors and families have little scientific information to decide which type of diaper to use. The present study aimed to describe features and habits of diaper area care and compare the frequency of diaper dermatitis in infants using cloth diapers with those using disposable diapers.

## Methods

The study was analytical and observational, with prospective data collection, approved by the institution's Human Research Ethics Committee (CAAE 57591022.5.0000.0096). Data collection took place from January to March 2022, with a research instrument sent via Google Forms® and prepared by the researchers. All participants signed an Informed Consent Form.

It included parents of children aged 5 years or less from any Brazilian state or Brazilians living abroad, who had not started the potty-training process and, therefore, used diapers daily. Participants who did not answer >50 % of the questions in the research instrument, and those with children over the age of 5, were excluded.

The research was publicized by the authors through the social media Instagram® and WhatsApp®. Participants were directed to 2 links: containing a questionnaire – one for the group that exclusively used disposable diapers, and another for those who used exclusively cloth diapers. After 60 days, information from the questionnaires (in Google Forms®) was extracted into Microsoft Excel® spreadsheets.

The diaper dermatitis diagnosis was based on parents' reports of mild or severe diaper rash injuries per year. Demographic information such as age, sex, and perineal hygiene habits, number of diaper changes, hygiene method, and use of diaper rash prevention ointments. A detailed description of the questionnaire is provided in the Supplementary Material.

Data were analyzed with the Statistica 4.0 Program (StatSoft Power Solutions, Inc., Palo Alto, California, USA). Continuous variables are expressed as mean (standard deviation) and median (interquartile range), while categorical variables are expressed in their absolute and relative frequencies. To estimate the difference between continuous variables of an asymmetric nature, the Mann-Whitney test was applied, while for categorical variables, the Pearson chi-square test was applied. The multivariate logistic regression model was applied to estimate the main factors associated with mild and severe diaper dermatitis, also illustrated in Forest Plot graphs. Residual deviation graphs indicated a homogeneous distribution, pointing to a well-adjusted model. The maximum value of the variance inflation factors (VIF) was 3, indicating the absence of multicollinearity. The univariate logistic regression model was used to estimate the association between the probability of mild diaper dermatitis and age.

## Results

**Figure 1:**
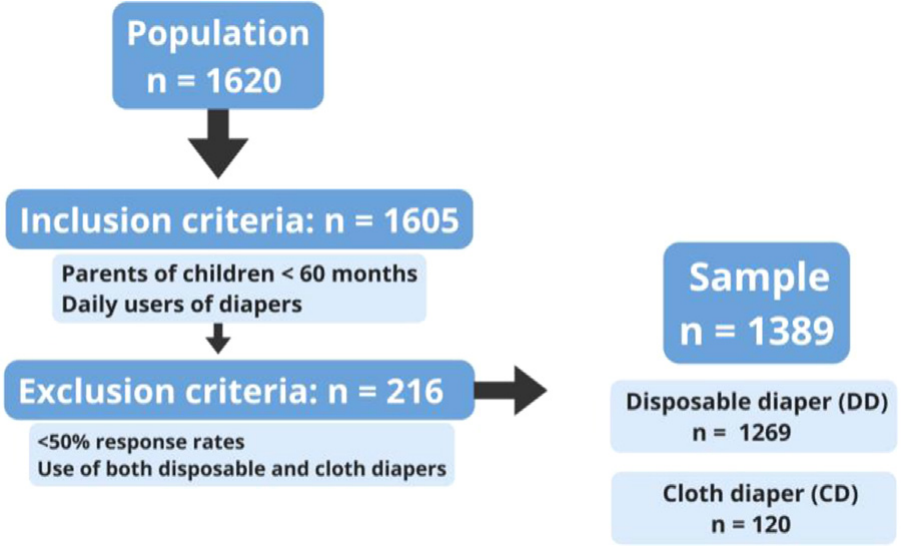
Diagram with number of participants in each group. DD, Disposable diaper group. CD, Cloth diaper group.

The study population comprised 1620 participants, of which 1605 met the inclusion criteria. Two hundred and sixteen were excluded, with a final sample of 1389 cases, among them, 1269 children (91.4 %) used disposable diapers (DD), and 120 (8.6 %) used cloth diapers (CD) (Figure 1).

The median age of the participants was 16 months (IIQ = 7–24 months), more precisely 16 months (IIQ = 8 = 24) for the DD group and 13 months (IIQ = 5–22) for the CD group (*p* = 0.03), with approximately 53.0 % of males in both groups (*p* = 0.94).

Around 80 % of the children used 5 to 8 diapers a day, ranging from 1 to 20 for the DD and 1 to 15 for the CD group (*p* = 0.52).

Regarding the cleaning method during diaper changes, in the DD there was a predominance of the use of wet wipes (61.5 %), while in the CD the use of cotton or cloth and water (62.2 %) prevailed (*p* < 0.001).

Barrier ointments were used by 68 % of children in the DD and 26.3 % in the CD (in some or all diaper changes); and in 28.7 % of the DD and 59.7 % of the CD they were used only in the presence of diaper rash (*p* < 0.001) (Table 1A).

**Table 1:** Cleaning methods and use of barrier ointments (A); Multivariate logistic regression for risk factors for diaper dermatitis (B).

Table 1A Cleaning methods and use of barrier ointments according to study groups.							
Cleaning methods Use of barrier ointments		DD (*n* = 1269)			CD (*n* = 120)	p	
Cleaning method							
Only cotton/ cloth and water		426 (37.4 %)			61 (62.2 %)	**< 0.001**	
Only wet wipes		700 (61.5 %)			29 (29.6 %)		
Both		13 (11 %)			8 (8.2 %)		
Barrier ointments							
Never		3.4 %			14.1 %	**< 0.001**	
Only if rash		28.7 %			59.7 %		
In some diaper changes		39.0 %			21.2 %		
In all diaper changes		29.0 %			5.1 %		

Table 1B Risk factors for diaper dermatitis.							
Factors	Mild diaper dermatitis				Severe diaper dermatitis		
	OR		IC 95 %	p	OR	IC 95 %	p
Sex	1.00		0.78–1.28	0.96	2.56	0.89–7.29	0.07
Age (months)	**1.92**		**1.39–2.66**	**<0.001**	**5.40**	**0.68–42.30**	**0.04**
Type of diaper	1.06		0.66–1.70	0.79	0.86	0.17–4.30	0.86
Number of diaper/ 24h	1.18		0.94–1.48	0.14	0.85	0.35–2.08	0.73
Wet wipes	1.16		0.92–1.48	0.19	0.51	0.20–1.32	0.17
Barrier ointments	0.96		0.72–1.26	0.77	0.72	0.25–2.02	0.53

Pearson chi-square test.

Bold font indicates statistical significance.

**Figure 2:**
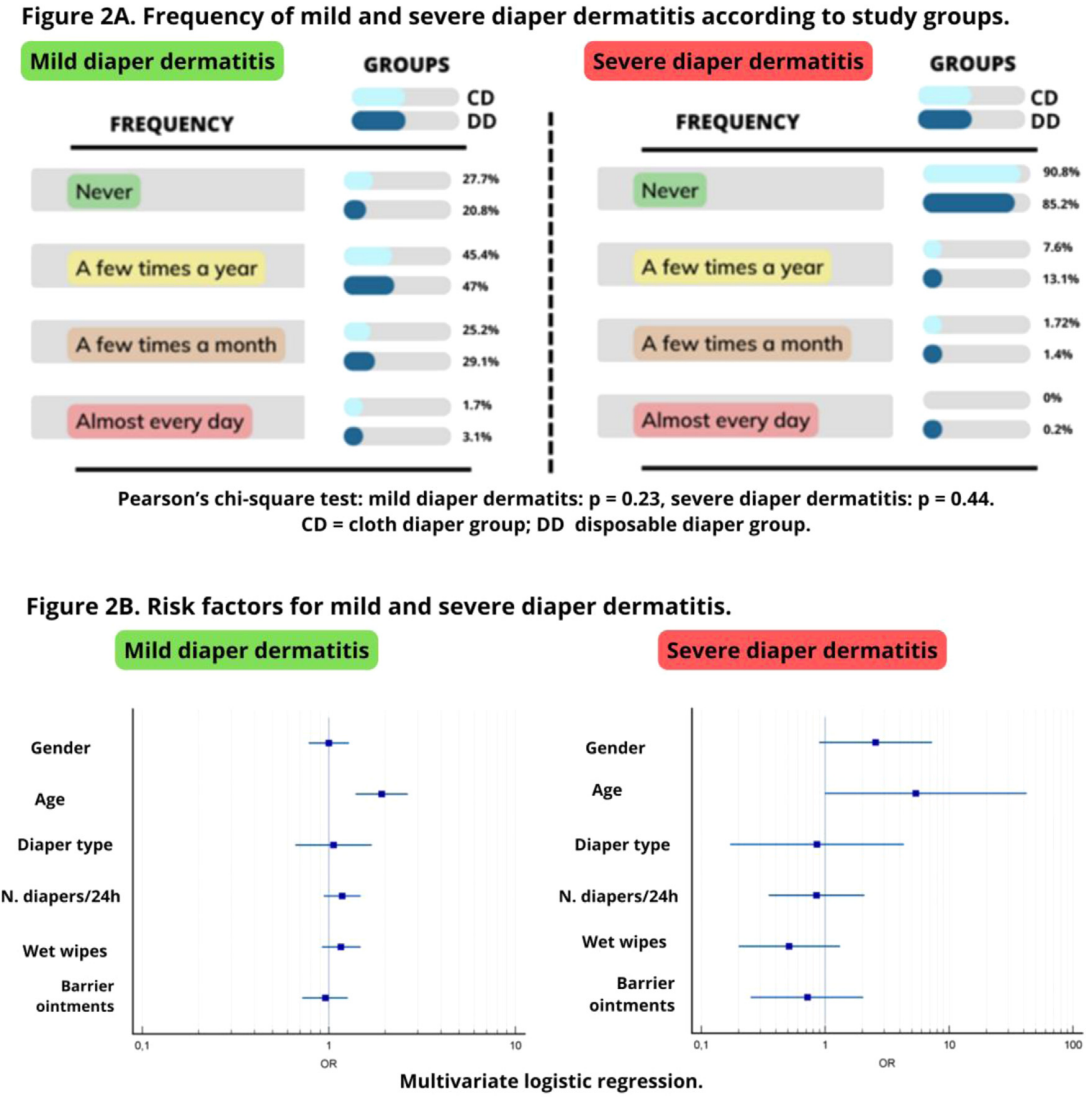
Frequency of Mild and Severe diaper dermatitis (A); Forest plot of risk factors for mild and severe diaper dermatitis (B). DD, Disposable diaper group; CD, Cloth diaper group.

In the multivariate analysis to identify predictive factors for mild diaper dermatitis, it was observed that age below 24 months increased the risk of diaper dermatitis by twofold (OR = 1.92, 95 % CI = 1.39–2.66, *p* < 0.001). For severe diaper dermatitis, the same factor was observed, with a five times higher risk associated with age (OR = 5.40, 95 % CI = 0.68–42.30, *p* = 0.04) (Table 1B) (Figure 2).

Mild diaper rash occurred a few times a year in 47.0 % and 45.4 % in the DD and CD groups, respectively (*p* = 0.47). Severe diaper rash occurred a few times a year in 13.1 % and 7.6 % in the DD and CD groups, respectively (*p* = 0.66). No significant difference was observed in the frequency of mild (*p* = 0.23) or severe (*p* = 0.44) diaper dermatitis between the study groups (Figure 2).

## Discussion

Cloth diapers have been used for infants for centuries, with the most diverse fabrics.^3^ In the 20th century, disposable diapers began to be produced and improved, making families' routines easier. From 1960 onwards, disposable diapers replaced cloth diapers.^5^

Disposable diapers are made of synthetic materials such as polypropylene and polyethylene, elastics, and adhesives. The inner layer is made of cellulose and absorbent polymer (polyacrylamide and/or sodium polyacrylate) which, when absorbing urine, forms a gel, preventing the liquid from returning to contact with the skin, without exaggerated increase in size (contributing to the infant's comfort). Optionally, their material may be impregnated by lotions that are released onto the skin after contact, promoting hydration and improving the skin barrier.^5^^,^^6^

Although disposable diapers are still the most frequent choice for families, cloth diapers have been increasingly used, whether for cost reasons or environmental concerns.

**Figure 3:**
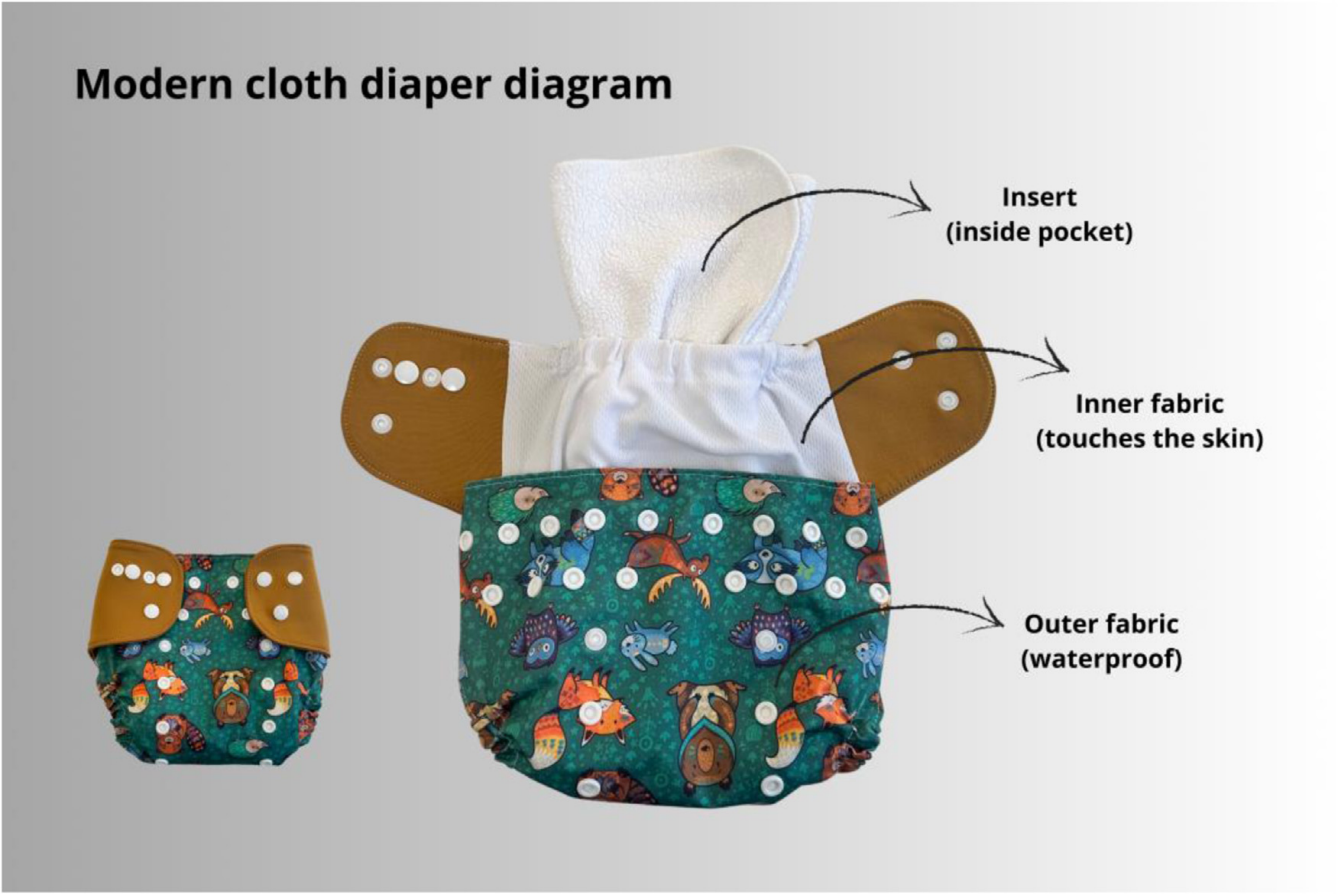
Image showing a cloth diaper diagram. Image authorized by the manufacturer, who kindly provided the cloth diaper, Malana Eco®.

Current cloth diapers are made up of layers.^7^ The top layer (in contact with the skin) is made up of fabrics that are permeable to urine (e.g. microfleece or suede), but do not retain moisture. To keep the skin dry, there is an absorbent internally positioned, usually made of melton (approximately 80 % cotton and 20 % polyester) or microfiber, in layers, adjusted to the size of the diaper pocket. Absorbents allow the diaper to be used for some hours, keeping the skin dry. The (outer fabric is a waterproof, breathable, and flexible cover, usually made of laminated polyurethane, lycra, tactel, or other fabrics. The cover is closed with adjustable buttons or velcro, which fits infants weighing 3 to 16–20 kg, so it can be used from newborns to potty training. The cloth diaper exemplified in Figure 3 is the “pocket” or “pocket/cover” type and is commonly used. There are other compositions, such as “all-in-one”, made with similar fabrics, but with the absorbent sewn into the inside of the diaper, in direct contact with the skin and which cannot be removed for washing (Figure 3).

After the feces have been removed, the ecological diapers can be machine-washed with regular soap. Drying is preferably done in the sun, ironing or tumble drying are not recommended. There is no need to wash dirty eco-friendly diapers daily. It is possible to store them in a waterproof bag or bucket with a lid for washing after a few days. To facilitate the removal of feces, disposable wipes called liners can be used between the skin and the diaper. They are generally biodegradable and can be disposed of along with feces in the toilet.

Cloth diapers in pediatric care have some disadvantages. They may require more frequent changes, when compared to disposable diapers, to prevent urine and stool leakages, depending on the number of absorbent cloth layers used. Thorough cleaning and sanitization are necessary to avoid bacterial contamination and reduce odor, potentially increasing parental workload.^3^

Diaper dermatitis is an irritant contact dermatitis on the perineal skin of newborns or infants. It is determined by contact with urine and feces in a constantly humid environment and under occlusion (diapers).^1^ It is not an allergic contact dermatitis to the type of diaper. Diaper dermatitis is identified as an erythema in the perianal, vulvar and thigh region - often sparing the inguinal folds - and is more common in infants aged 6 months to 2 years.^4^ Skin irritation is caused by an increase in the pH of the skin, caused by an increase in the activity of enzymes that convert urea into ammonia, and skin friction during hygiene when changing diapers is a worsening factor.^5^

The increase in the frequency of use of disposable diapers over the years has been related to a significant reduction in the incidence of diaper dermatitis, a fact attributed to absorbent materials that reduce the contact of excreta with the skin,^5^ although there is no proof of the greater effectiveness of one or another diaper material.^8^

There are cases of diaper dermatitis with the use of the old types of cloth diapers.^5^ Harfmann et al., 2017, reported 4 infants using cloth diapers, without specifying the type, with vesico-bullous lesions in the perineal region that were refractory to usual treatments for diaper dermatitis, and that improved with the change to disposable diapers or potty training.^9^ In 1982, Stein and Brook evaluated 200 infants in a controlled and blind manner, divided into 4 groups: 1 using common cloth diapers (without specifying the type of fabric) with domestic washing, and another 3 using disposable diapers. The incidence of diaper dermatitis in the cloth diaper group was higher.^10^ Babu et al., 2015, evaluated 253 babies in a neonatal intensive care unit divided into 2 groups – one using 3-layer disposable diapers, the other using cotton diapers as a single layer (white cotton fabric folded in a triangular shape). The disposable diaper group had a significant reduction in the incidence of probable sepsis. However, this study considered the use of reusable diapers made with only one layer of cotton, as a single fabric,^9^ different from the reusable diapers currently used, composed of more than one fabric and layer.

Liu et al., from the company Procter and Gamble, described an evaluation carried out by nurses including 694 Chinese children using traditional cloth diapers exclusively (single fabric held on the participant by an elastic band).^11^ The intention of using this type of diaper in the Chinese population is to indicate that there has been urination or evacuation for an immediate diaper change, so waterproof materials are not commonly used. Therefore, diaper changes during the day and night are more frequent with Chinese cloth diapers. Healthy babies aged 3 to 9 months using exclusive diapers, at least in the last 7 days, were included. Sixty percent of the infants had undergone at least 3 diaper changes the night before the visit. There was no rash or redness on the skin of the genital region and buttocks in 76.5 % of the infants, but 51.4 % had lesions in the intertriginous areas and 70.6 % had some degree of perianal dermatitis. The authors concluded that this type of diaper does not increase the frequency of diaper dermatitis in convex areas, but rather perianal and intertriginous areas.^11^

Maruani et al., 2013, reported 5 infants aged 7 to 17 months using reusable diapers for at least 6 months, with severe perineal dermatitis for weeks to months, which improved with the use of disposable diapers and topical treatments.^4^ These patients presented ulcerated or papulonodular lesions in the perianal and genital regions (convex regions). In 3 of the 5 cases of skin biopsy, there was nonspecific inflammation, such as hyperkeratosis and perivascular inflammatory infiltrate in the superficial dermis. The authors conclude that reusable diapers were less absorbent than disposable diapers, which contributed to prolonged contact of the skin in the convex regions with urine and feces. As a result, fecal enzymes damage the skin and alter the pH of the region.^4^

A cross-sectional study with 1153 Thai children aged 1–24 months, by Sukhneewat et al., identified as significant risk factors for diaper dermatitis: <3 nightly diaper changes, use of cloth diapers (although without specifying the type), use of talcum powder in the diaper region, and previous episodes of diaper dermatitis.^12^

Although the cited studies indicate a higher frequency of diaper dermatitis in babies who use reusable diapers, most of them do not consider the new currently used type of reusable diapers, made up of layers that simulate disposable diapers. In the present study, the frequency of diaper rash was similar among infants using cloth and disposable diapers. Another point to consider is that the ED group used diaper rash prevention ointments less frequently than the DD group. Since these ointments help prevent diaper dermatitis, it is plausible to consider that ecological diapers were more effective than disposable diapers as protective factors against DCD.

Regarding the hygiene diaper changing method, it was observed that in the DD group, the wet wipe was the most used. In the CD group, cleaning with cotton/cloth and water was more frequent – which matches the profile of families who seek to use more natural materials and substances on their children's skin. The use of wet wipes can also influence the frequency of diaper dermatitis, since they can contain substances that might irritate the skin, such as alcohol, sodium lauryl sulfate, methylisothiazolinone (MI), methylchloroisothiazolinone and perfume.^13^ Even the DD using more wet wipes had diaper dermatitis at the same frequency as the CD, possibly due to using more prevention ointments. These data demonstrate the multifactorial nature of diaper dermatitis and the importance of clarifying all aspects of hygiene and care in the diaper area with the guidance of families.

The present study found that for the family profiles included, only an age of <24 months was identified as a significant risk factor for diaper rash. Other factors such as the use of wet wipes or diaper rash ointments, although showing significant differences in usage frequency between groups, did not have an impact on the incidence of diaper rash. It is important to note that daily care habits for the diaper area, including cleaning methods and skin protection, are influenced not only by the objective frequency of diaper rash but also by cultural patterns and personal consumption preferences.

Environmentalists argue that reusable diapers are less harmful to the environment.^14^ Disposable diapers make up almost 2 % of the total weight of urban solid waste generated in Brazil.^15^ Besides, during the manufacturing process of disposable diapers, plastics, polymers, tapes, elastics, and adhesives are synthetic products produced from naphtha, which is a fraction of petroleum, known as a non-renewable raw material.^6^^,^^15^

On the other hand, the disposable diaper industry points out, for example, in a 2011 Procter and Gamble laboratory review, that cloth diapers consume energy, water, detergents, and machinery for their washing and, therefore, their impact is comparable to the impact caused by disposable diapers, discarded in the trash.^5^ However, this quote is not based on clear studies that demonstrate this information in numbers.

The author Stephen Leahy states, in his book “Your Water Footprint”, 2014, that a disposable diaper consumes an average of 545 liters of water to be produced. If a baby uses approximately 6000 disposable diapers in total, there is a total consumption of 3.27 million liters of water.^14^ To produce a cloth diaper, an average of 15 liters is consumed. A common washing machine uses an average of 140 liters per cycle. If used 3 times a week for 3 years, the total water consumption for washing would be 65.5 thousand liters^14^ - significantly lower than the consumption generated by disposable diapers.

The limitations of the present study include differences in the content of the questions between the groups and a possible memory bias due to remote answering of questions. The study also lacked a physical examination, which could have included other diagnoses of diaper area diseases. Additionally, the absence of information on the subject's health, such as illness incidence, could have influenced the incidence of diaper rash. The study did not have a consistent pattern in the types of diapers used, which could have affected the frequency of diaper dermatitis. Lastly, there was a significant difference in the number of participants between the groups, with disposable diapers being more commonly used, but the number of exclusively cloth diaper users was remarkable compared to other studies.

Cloth diapers currently used by the Brazilian population are effective in containing excreta and reducing their contact with children's skin. They present a risk of diaper dermatitis at a similar frequency to disposable diapers, contrary to what is indicated in the literature based on case reports or studies that do not specify the type of reusable diaper used, or that used old cloth diapers (in a single layer) that do not reduce the contact of urine and feces with the skin.

## Conflicts of interest

The authors declare no conflicts of interst.

## Appendix Supplementary materials

Image, application 1
