# Supplementary material for: Cloth versus disposable diapers: an exploratory study on family habits
Source: J Pediatr (Rio J). 2024 Dec 9;101(2):276–81. doi: 10.1016/j.jped.2024.10.008 (PMC11889673; doi:10.1016/j.jped.2024.10.008)
Supplement: Supplementary file 1 [file mmc1.docx]

**JPED-D-24-00115 – Suplemmentary Material**

SUPLEMMENTARY MATERIAL

1. Questionnaire applied to families who only use disposable diapers:

| Guidelines:  Please answer considering 1 CHILD PER QUESTIONNAIRE  You can mark more than one answer  This questionnaire should only be answered in the case of children who have not yet started potty training (whether daytime or nighttime)  How old is your baby (in months)? ____  What is your baby's sex? ___ Feminine ___ Masculine  What is the highest level of education of the parents or legal guardians?  Incomplete elementary education  Complete primary education  Complete high school  Complete higher education  Complete postgraduate degree  How many diapers (in total) do you use on average during a day (24 hours)? ______  The brand(s) of disposable diapers you use are: ________________________  How do you clean the skin during diaper changes most of the time?  Cotton + water with or without soap  Wet wipe  Cotton and Cleansing water or cleansing lotion  Running water  Other: ______________________  Do you use ointments to prevent diaper rash? (e.g. weleda, bepantol, desitin, hypoglos, babymed, etc.)  Never  Only if you have diaper rash  In the minority of diaper changes  Most diaper changes  At every diaper change  Do you use oils to prevent diaper rash? (e.g. sunflower oil, Dersani, olive oil, etc.)  Never  Only if you have diaper rash  In the minority of diaper changes  Most diaper changes  At every diaper change  Does your baby have a diaper rash now?  ___ Yes ___ No  Your baby has had mild diaper rash (redness of the skin around the diaper)  ( ) Never  ___ times a year  ___times per month  ___times a week  ( ) Almost every day  Has your baby ever had severe diaper rash that needed to be treated by a healthcare professional?  ( ) Never  _____ times a year  _____times a month  _____times a week  ( ) Almost every day  Have you ever met a family who uses reusable diapers?  No  Yes |
| --- |

1. Questionnaire applied to families who only use cloth diapers:

| **Guidelines:** Please answer considering 1 CHILD PER QUESTIONNAIRE  You can mark more than one answer  This questionnaire should only be answered in the case of children who have not yet started potty training (whether daytime or nighttime) |
| --- |
| How old is your baby (in months)? ____  What is your baby's sex? ___ Feminine ___ Masculine  What is the highest level of education of the parents or legal guardians?  Incomplete elementary education  Complete primary education  Complete high school  Complete higher education  Complete postgraduate degree  Do you use reusable diapers (e.g. fabric) on your baby(ren)?  Yes, reusable only  Yes, most of the time  Only rarely (usually uses disposables)  No, only use disposables  How many diapers (in total) do you use on average during a day (24 hours)? ______  What type of reusable diapers do you use?  "Pocket" type: separate absorbent, inside the cover.  "Cover" type: absorbent placed in contact with the skin.  "All in 1" type: absorbent sewn inside the lining (inside the pocket)  Common cloth type with plastic pants  Other: ____________  What fabric is the inner part of your reusable diaper (lining or pocket) made of, the one that comes into contact with the skin?  Cotton  Soft  Dry fit  Suedine  Other: ____________  It's the absorbent itself (I use a "cover" type diaper)  I don't know  The brand(s) of reusable diapers you use are:  Chinese  Brazilian - which one?: ________________________  I don't know  What fabric are the reusable diaper pads you use most of the time?  Synthetic (microfiber, fleece, polyester, polyamide)  Melton/Unifloc  Hemp or hemp  Bamboo  Bamboo Charcoal  Cotton (creamer type)  Other: ___________  Do you use liners in diapers?  Ever  Sometimes  Rarely  Never  What type of liner is used most of the time?  Reusable  Biodegradable disposable  Non-biodegradable disposable  Other disposable:  Other: _________  How do you wash reusable diapers?  In the machine  Manually  What soap do you use for common washing?  Bar soap  Washing powder  Liquid soap  Other: __________________  In addition to soap, do you use any other products for washing?  Sodium percarbonate (e.g. Vanish)  Sodium bicarbonate  Vinegar  Bleach  Tea tree essential oil  Disinfectant  Salt  Shampoo anti residue  Other? _______________  How often (approximately) do you use the product(s) mentioned above?  In every wash  1x per week  Fortnightly  1x per month  1x every 2 months  1x every 3 months  Other: ______________  Do you do "residual"/"deep" washing?  No  Yes  How do you deep wash?  With hot water  With bleach  Other: ___________  How often do you deep wash?  Fortnightly  1x per month  1x every 2 months  1x every 3 months  Other: ______________  Do you usually soak pads before washing?  No  Yes  With which product?  Sodium percarbonate (e.g. Vanish)  Sodium bicarbonate  Vinegar  Bleach  Tea tree essential oil  Disinfectant  salt  Shampoo anti residue  Other? _______________  How do you clean your skin during diaper changes most of the time?  Cotton + water with or without soap  Wet wipe  Cotton and Cleansing water or cleansing lotion  Running water  Other:  Comments:  Do you use ointments to prevent diaper rash? (e.g. weleda, bepantol, desitin, hypoglos, babymed, etc.)  Never  Only if you have diaper rash  In the minority of diaper changes  Most diaper changes  At every diaper change  Do you use oils to prevent diaper rash? (e.g. sunflower oil, Dersani, olive oil, etc.)  Never  Only if you have diaper rash  In the minority of diaper changes  Most diaper changes  At every diaper change  Does your baby have diaper rash (redness in the diaper area) now?  ___ Yes ___ No  Does your baby have mild diaper rash (redness of the skin around the diaper)? -- CONSIDER THE AGE OF THE CHILDREN??  ( ) Never  _____ times a year  _____times a month  _____times a week  ( ) Almost every day  Has your baby ever had severe diaper rash that needed to be treated by a healthcare professional?  ( ) Never  _____ times a year  _____times a month  _____times a week  ( ) Almost every day  What was your motivation for using reusable diapers?  Money saving  Environmental concern  Less chance of diaper rash  Other: ____________  How satisfied are you with using reusable diapers?  I like it a lot, I recommend it  I like  I didn't like it, but I use it to avoid losing my investment.  I don't like it, I plan to stop using it  Would you use reusable diapers on another child, if you had one?  Yes  Perhaps  No |
|  |
|  |
